# Dysregulation of SWI/SNF Chromatin Remodelers in NSCLC: Its Influence on Cancer Therapies including Immunotherapy

**DOI:** 10.3390/biom13060984

**Published:** 2023-06-13

**Authors:** Yijiang Shi, Daniel Sanghoon Shin

**Affiliations:** 1 Division of Hematology/Oncology, Department of Medicine, Los Angeles, CA 90073, USA; yjshi@ucla.edu; 2 Division of Hematology/Oncology, Department of Medicine, VA Greater Los Angeles Healthcare System, 11301 Wilshire Blvd, Los Angeles, CA 90073, USA

**Keywords:** SWI/SNF, biomarker, immunotherapy, NSCLC

## Abstract

Lung cancer is the leading cause of cancer death worldwide. Molecularly targeted therapeutics and immunotherapy revolutionized the clinical care of NSCLC patients. However, not all NSCLC patients harbor molecular targets (e.g., mutated EGFR), and only a subset benefits from immunotherapy. Moreover, we are lacking reliable biomarkers for immunotherapy, although PD-L1 expression has been mainly used for guiding front-line therapeutic options. Alterations of the SWI/SNF chromatin remodeler occur commonly in patients with NSCLC. This subset of NSCLC tumors tends to be undifferentiated and presents high heterogeneity in histology, and it shows a dismal prognosis because of poor response to the current standard therapies. Catalytic subunits SMARCA4/A2 and DNA binding subunits ARID1A/ARID1B/ARID2 as well as PBRM1 were identified to be the most commonly mutated subunits of SWI/SNF complexes in NSCLC. Mechanistically, alteration of these SWI/SNF subunits contributes to the tumorigenesis of NSCLC through compromising the function of critical tumor suppressor genes, enhancing oncogenic activity as well as impaired DNA repair capacity related to genomic instability. Several vulnerabilities of NSCLCS with altered SWI/SNF subunits were detected and evaluated clinically using EZH2 inhibitors, PROTACs of mutual synthetic lethal paralogs of the SWI/SNF subunits as well as PARP inhibitors. The response of NSCLC tumors with an alteration of SWI/SNF to ICIs might be confounded by the coexistence of mutations in genes capable of influencing patients’ response to ICIs. High heterogenicity in the tumor with SWI/SNF deficiency might also be responsible for the seemingly conflicting results of ICI treatment of NSCLC patients with alterations of SWI/SNF. In addition, an alteration of each different SWI/SNF subunit might have a unique impact on the response of NSCLC with deficient SWI/SNF subunits. Prospective studies are required to evaluate how the alterations of the SWI/SNF in the subset of NSCLC patients impact the response to ICI treatment. Finally, it is worthwhile to point out that combining inhibitors of other chromatin modulators with ICIs has been proven to be effective for the treatment of NSCLC with deficient SWI/SNF chromatin remodelers.

## 1. Introduction

Lung cancer is the leading cause of cancer death worldwide. In 2022, more than thirteen thousand patients died of lung cancer in the United States alone [1]. The poor prognosis is attributed to the high morbidity and low response rate to conventional chemotherapy because the majority of patients with lung cancer are only diagnosed at an advanced stage. Further, 85% of lung cancers are categorized by their histological types as a non-small cell lung cancer (NSCLC) subtype [2].

Targeted therapy revolutionized the clinical care of NSCLC patients. Compared with conventional chemotherapy, which kills all the dividing cells, including normal cells, indiscriminately, targeted therapy selectively kills only cancer cells due to their dependence on these “driver” oncogenes to survive and proliferate [3].

Three of the most common actionable driver oncogenes in NSCLC are epidermal growth factor receptor (EGFR), anaplastic lymphoma kinase (ALK), and rat sarcoma virus (RAS) [4]. Since the initial approval of the first epidermal growth factor receptor (EGFR) inhibitor in 2003, three generations of EGFR TKIs have been developed for EGFR-dependent NSCLC [5]. Similarly, three generations of small molecular inhibitors targeting ALK have also been approved as the first-line treatment of ALK-positive advanced NSCLC [6]. Recently approved sotorasib and adagrasib are the first KRAS G12C mutation-targeting drugs for the treatment of NSCLC patients [7].

Screening for these driver genes via NGS (next-generation sequencing) has become standard care for patients with NSCLC [8]. However, the majority of patients with NSCLC do not harbor known driver oncogenes and, therefore, targeted therapy is not an option for them. In addition, most patients will develop acquired resistance to targeted therapies, which further limits the value of targeted therapy for long-term disease control in NSCLC patients [9].

Cancer immunotherapy using immune checkpoint inhibitors (ICIs) has further supplemented the treatment regimen for NSCLC over the past decade.

Immune checkpoint proteins help cancer cells evade immune clearance by suppressing their native immune responses. ICIs block these negative signals from immune checkpoint proteins and reactivate cytotoxic T cells to eradicate cancer cells.

The activation of the adaptive immune response against cancer cells can be separated into two phases: the priming phase and the effector phase. During the priming phase in the lymph node, CD8+ T cells will be activated and differentiated into CD8+ cytotoxic T cells by the MHC II-bound tumor antigen. The priming phase of T-cell activation is mediated by the interaction of the T-cell receptor and the CD28 receptor with the class II major histocompatibility complex and the B7 co-stimulatory molecule located on the antigen-presenting cells. The interaction of cytotoxic T-lymphocyte-associated antigen 4 (CTLA-4), an immune checkpoint protein, with the B7 molecule delivers an inhibitory signal to suppress T-cell activation during the priming phase. During the effector phase of the tumor, activated CD8+ cytotoxic T cells attack cancer cells upon recognition of tumor antigens presented by MHC I molecules. At the same time, activated CD8+ cytotoxic T cells will induce increased expression of the programmed cell death ligand (PD-L1) protein on the surface of the cancer cells through the release of cytokines such as IFN-γ. The PD-L1 protein will bind with the programmed cell death-1 (PD-1) protein expressed on activated T cells to suppress T-cell-mediated attacks on cancer cells.

Accordingly, ICIs can be divided into two categories, i.e., antibodies targeting the PD-1/PD-L1 axis that mainly act on the immune effector phase and antibodies targeting CTLA-4 that act on the immune-priming phase [10]. Table 1 lists all the ICIs that have been approved by the FDA for NSCLC treatment. Nivolumab, pembrolizumab, and cemiplimab are antibodies targeting the PD-1 receptor. Atezolizumab and durvalumab are PD-L1 antibodies. Inhibition of PD-1/PD-L1 blocks the interaction of PD-L1 with the PD-1 proteins and releases the inhibition of immune responses towards tumors. Ipilimumab and tremelimumab are antibodies targeting the CTLA-4 protein. Ipilimumab or tremelimumab prevents CTLA protein on the CD8+ cytotoxic T cells from binding with the B7 co-stimulatory protein on the antigen-presenting cell and, therefore, increases the amount of B7 protein capable of interacting with CD28, resulting in B7-CD28-mediated T-cell activation [11]. ICIs have become the standard of care for the treatment of advanced NSCLC as combinatorial immunotherapy, with or without chemotherapy [12].

ICIs have greatly improved the overall survival of a subset of NSCLC patients. However, the majority of NSCLC patients do not respond very well (because of primary resistance), and a substantial portion of patients who do respond initially will eventually develop acquired resistance [13]. Several outstanding questions are imperative for the field to answer, such as: (1) the mechanisms of response and resistance to immunotherapy; (2) the identification of biomarkers to predict response or resistance; and (3) how to overcome this resistance. The field has been uncovering the mechanisms of resistance, such as inadequate neo-antigens expressed in the tumor cells, impaired processing and presentation of tumor antigens to the T lymphocytes, limited T-lymphocyte infiltration into the TME (tumor micro-environment), compromised function of effector T cells by impaired interferon signaling, proficient immune suppressive cells, T-cell exhaustion, etc. [14]. We have been discovering the factors related to the response as well; some of them were evaluated as predictive biomarkers in clinical studies, such as PD-L1 expression (tumor and immune cells), high tumor mutational burden (TMB), and mismatch repair deficiency or microsatellite instability in the tumor biopsy. Although these markers are able to guide clinical decision making, they are deemed incomplete, and it has become apparent that single markers cannot adequately represent the tumor biology in relation to response or resistance [15]. Hence, a comprehensive understanding of tumor biology, the tumor microenvironment, and other host factors, such as the microbiome, will be required for the accurate prediction of prognosis or response to immunotherapy [16]. Emerging studies suggest that epigenetics, particularly chromatin regulators, play a significant role in tumor biology and the functions of immune cells in TME [17].

In this review, we will first briefly discuss the basic biology of cancer epigenetics, followed by a discussion of the composition and module structures of human SWI/SNF chromatin. Then, we will review the recent findings about the mechanisms by which deregulation of SWI and SNF subunits contributes to the tumorigenesis of NSCLC. Next, we will discuss the vulnerability and strategies for targeted therapies for NSCLC with deregulation of SWI/SNF complexes. Lastly, we will discuss the relationship between the efficacy of the ICI treatment and the loss of the human SWI/SNF complex in patients with NSCLC.

## 2. Cancer Epigenetics and SWI/SNF Chromatin Remodelers

Dysregulated gene expression is critical for the initiation and progression of all malignancies [18]. In eukaryotes, DNA is tightly compacted with histone proteins in the nucleus. Its length is condensed from the original 2 m to as short as 10 μm to fit into the nucleus. The complex, consisting of DNA and histone proteins, constitutes chromatin. The basic structure of chromatin is a nucleosome: a 146 bp DNA fragment wrapped around a histone octamer consisting of an H2A dimer, an H2B dimer, and an H3 and H4 tetramer attached with two H1 globular proteins and various lengths of linker DNA. Multiple nucleosomes in the DNA present a “beads on a string” appearance. The nucleosome forms the 30 nM fiber secondary structure of chromatin, which compacts further into higher-order chromatin structures hierarchically [19]. The compactness of the chromatin obstructs regulatory proteins from accessing DNA, which is essential during biological processes, such as DNA transcription, DNA replication, and DNA-damaging repairs. Therefore, chromatin structure, especially the distribution of nucleosome over DNA, needs to be regulated dynamically to maintain an appropriate “openness” of DNA for various regulatory proteins [20].

There are three major chromatin regulators: covalent chromatin regulators, non-coding RNAs, and non-covalent chromatin regulators. They work together to determine a specific chromatin structure in a cell. Covalent chromatin regulators regulate DNA methylation and demethylation, histone acetylation and deacetylation, histone methylation and demethylation, etc. Non-coding RNA is involved in the process of histone modification [21]. Non-covalent chromatin regulators regulate nucleosome sliding, nucleosome ejection, nucleosome assembly, nucleosome editing, and variant histone replacement. There are four non-covalent chromatin regulators: switch/sucrose non-fermentable (SWI/SNF), imitation switch (ISWI), chromodomain helicase DNA binding (CHD), and INO80. They are evolutionarily conserved chromatin remodeling complexes that have different subunit compositions, and each plays a non-redundant role in executing ATP-dependent chromatin remodeling [22].

Covalent chromatin regulators change marks on the chromatin by increasing (“writing”) or decreasing (“erasing”) post-translational modifications of histones. These marks on the histone (histone hint) will be recognized (“read”) by non-covalent chromatin regulators.

Therefore, the specific chromatin structure of each cell, i.e., the epigenome, is the result of collaboration among different chromatin regulators. The epigenome determines which genes in the genome will be expressed. Similar to the genetic phenotype, the epigenome of a cell can be inherited from a parent cell to its descendent cells. In contrast to the genetic regulation, the epigenome of a cell is not stored in DNA. A unique epigenome exists in each cell, depending on the differentiation, development, and proliferation status of the cell, while all of the cells should contain the same DNA sequence, as long as they are from the same entity.

Mutations and deregulation in the epigenetic regulators happen frequently in malignancies. For example, the overexpression of DNA methyltransferases (DNMTs) and histone deacetylases (HDACs) is frequently found in various malignancies. Targeting epigenetic regulators for cancer therapeutics, especially for the treatment of hematological malignancies, has attracted extensive attention. At least nine drugs targeting epigenetic regulators have been approved by the FDA for the treatment of hematological malignancies [Table 2], and their potential for the treatment of solid cancers has been investigated in clinical trials [17]. It is noteworthy that hydralazine, previously approved for the treatment of hypertension [23], has been shown to have inhibitory properties on DNA methylation [24] and has been tested for the treatment of prostate cancer [25].

Alterations in chromatin remodeling complexes are commonly found in many malignancies [20], and the frequency of alterations or mutations is the highest in the SWI/SNF chromatin remodeling complex. It is possibly related to the unique ability of the SWI/SNF complex to slide or eject nucleosomes from chromatin, rendering chromatin more accessible for regulatory proteins and RNA. It has been found that 20% of human malignancies contain at least one mutation in the subunits of SWI/SNF chromatin remodelers [27].

## 3. The Composition and Modular Structure of Human SWI/SNF Chromatin Remodelers

SWI/SNF chromatin remodelers are macromolecular complexes assembled by various subunits. These subunits are encoded by 29 different genes. Each SWI/SNF complex consists of 10 to 15 subunits. Discrete combinations of various subunits together with multiple splicing variances of the subunits generate a great number of different SWI/SNF chromatin remodelers. Each SWI/SNF complex contains one mutually exclusive catalytic subunit, either SMARCA4 or SMARCA2 (with an alias of BRG1 or BRM, respectively). Thus, the SWI/SNF complex is also called the BRG1/BRM-associated factor (BAF). Depending on the different combinations of the subunits, human BAFs are classified into canonical BAF (cBAF), polybromo-associated BAF (PBAF), and noncanonical BAF (Table 3).

The SWI/SNF complexes frequently bind to the enhancers and promoters of their targets. All the SWI/SNF complexes utilize energy provided by the catalytic subunit through ATP hydrolysis to remodel chromatin structure through nucleosome sliding and eviction mechanisms [32].

The recent results of the cryo-electron microscopy studies suggest that all the subunits in the human SWI/SNF complex are organized into a similar “clamp”-like three-module structure to interact with their subject, the nucleosome [32]. For example, the cBAF complex consists of the adenosine triphosphatase (ATPase) module, the actin-related protein (ARP) module, and the base module. Within the ATPase module, the C-terminal of SMARCA4 grasps the nucleosome. The ARP module bridges between the ATPase module and the base module. Within the base module, SMARCB1 binds to the nucleosome, and ARID1A/B stabilizes the base module by binding to SMARCB1, the N-terminal of SMARCA4, and all other base units, which is required for efficient nucleosome sliding activity of the cBAF [33].

Subunits in the PBAF complex are organized into a tripartite modular structure, just like those in the cBAF complex. The three modules of the PBAF complex are the ATPase module, the ARP module, and the substrate recruitment module (SRM). The ATPase module and ARP module of the PBAF play similar functions as in the cBAF complex. As a homolog of ARID1A/B in cBAF, ARID2 is essential to the activity of PBAF to slide nucleosomes. Differently from cBAF, there are three unique histone binding subunits, PHF10, PBRM1, and BRD7, in PBAF, forming a submodule [34]. The PHF10 subunit binds to the histone tails through its plant homeodomain (PHD) fingers, recognizing methylated and unmethylated histone H3K4; the BRD7 subunit binds to the histone tail through the bromodomain, recognizing the acetylated lysine residues of histone; PBRM1 binds to histone tails through a total of six bromodomains, recognizing acetylated lysine residuals of histones; and the bromo adjacent homology (BAH) domain, recognizing methylated histones and nucleosomes [28,29,34,35,36]. Results of the cryo-electron microscopy studies in the mammalian cell also suggest that a similar module structure exists in human ncBAF. In contrast to cBAF and PBAF, no ARID domain subunits exist in ncBAF. The location and function of ARID-containing subunits are replaced by the GLTSCR1/1L subunits [32]. The elucidation of the structure of the SWI/SNF complexes will help in studying the importance of disease-associated mutations in the different subunits.

## 4. The Most Common Dysregulation of SWI/SNF in NSCLC

The composition of SWI/SNF chromatin remodelers is tissue- or cell-type-specific [37]. Results from many large-scale exome-wide sequencing studies showed that different tumor types exhibit specific SWI/SNF mutation patterns [38]. For example, almost all malignant rhabdoid tumors (MRTs) had an inactivated mutation of the SMARCB1 gene [39,40], and it was the only mutated SWI/SNF subunit found in MRT. To explore which SWI/SNF chromatin remodeler subunits are most likely altered in NSCLC, we queried 29 genes encoding for all the subunits of human SWI/SNF complexes for mutations in the NSCLC patients on the cBio Cancer Genomics Portal (http://cbioportal.org) dataset. Thus, 8854 patients and 11037 samples from 28 studies were included. The pool of NSCLC patients in the query covers all types of NSCLC, including squamous cell carcinoma, lung adenocarcinoma, and large-cell carcinoma. The top six subunits with the highest mutation frequencies are SMARCA4/2 (7% and 2.8%), the mutually exclusive catalytic subunits, ARID1A/ARID1B/ARID2 (6%, 4%, and 4%), the AT rich interactive domain-containing subunits, and PBRM1 subunits (2.5%) (Table 4).

Further classification of the type of mutation in the top six subunits showed that more than 40% of the mutations were missense, which could either positively or negatively influence the expression of the gene; SMARCA4, ARID1A, and ARID2 had the highest frequency of truncating mutations at 32.6, 50.8%, and 39.1%, respectively, which was usually correlated with the loss of protein expression. The result of our query showed that mutations of SMARCA4/A2, ARID1A/ARID1B/ARID2, as well as PBRM1 were the most common mutated SWI/SNF subunits in the population of NSCLC patients. These subunits are either catalytic subunits or histone/DNA-binding subunits. Loss of these subunits directly dampens the function of the SWI/SNF complexes in regulating the chromatin structure. The result was in agreement with that found in the literature [41,42,43]. ARID1A and ARID1B are highly homologous, mutually exclusive subunits in cBAF that can directly bind DNA. In PBAF, ARID2 and PBRM1 play similar roles in binding DNA. BRG1 and BRM are the only two mutually exclusive catalytic subunits. Their helicase ATPase domains provide energy for the sliding and eviction of nucleosomes from specific regions on the chromatin. cBAF has either BRG1 or BRM subunits. In addition, ARID1A, ARID2, BRG1, and PBRM1 are all bona fide tumor suppressors [27,38,44,45].

## 5. Mechanisms of Dysregulation of SWI/SNF Complexes Contributing to the Tumorigenesis of NSCLC

SWI/SNF complexes are master regulators of gene expression. They usually bind to the regions of the genome containing critical cis-acting transcriptional regulatory elements, such as enhancers and promoters [46]. cBAFs are mainly found in the chromatin region with enhancers, while PBAFs are found in the region of the proximal promoter. Dysregulation of the regulatory role of SWI/SNF complexes on transcription dramatically shifts the transcriptome landscape of the cell, leading to abnormal differentiation and the development of multiple cell types [47,48], irregular cell cycle regulation [49], and compromised DNA-damaging repair processes [28].

Therefore, the consequences of the dysregulation of SWI/SNF complex-induced alterations of chromatin structure are profound, from neurological disorders to the death of embryos as well as tumorigenesis. A definitive answer to explain how exactly the dysregulation of these SWI and SNF subunits leads to tumorigenesis is not available [41]. However, the research findings relating the alteration of SWI/SNF complexes to decreasing function of some tumor suppressor genes and increased expression of some oncogenes, as well as weakened DNA damage repair, may provide some clues about the mechanisms of dysregulation of SWI/SNF complexes contributing to the tumorigenesis of NSCLC [41,50,51].

### 5.1. Decreased Function of Tumor Suppressor Genes via the Dysfunction of SWI/SNF Complexes

The active status of enhancer elements of multiple tumor suppressor genes is maintained by the binding of SWI/SNF complexes [52]. Depletion of SWI/SNF complexes represses the expression of these genes. For example, loss of ARID1A was accompanied by decreased expression of several classical tumor suppressors, such as PIK3IP1 [53], CDKN1A [54], TGF-b [54], SMAD3 [55], and E2F4 [56]. In addition, the cell cycle regulation and cell growth inhibition actions of P53 and RB proteins depend on the SWI/SNF complex [57,58,59,60]. BRG1 and BRM can directly bind to the RB gene, facilitating its downstream targets responsible for cell cycle regulation and repression [61].

### 5.2. Increased Expression of some Oncogenes Caused by the Dysfunction of SWI/SNF Complexes

c-Myc is a transcription factor that is involved in almost every aspect of the oncogenic process, such as facilitating cell cycle regulation, proliferation, and differentiation of cancer cells. Loss of BAG11 enhances the activity of the c-myc gene, which contains a super-enhancer structure in human neuroblastoma cells [62,63] and melanoma cells [64]. Super-enhancers were described as a class of regulatory regions with unusually strong enrichment for the binding of transcriptional co-activators [46]. While SWI/SNF complexes repress acetylation in common enhancers, they prevent hyperacetylation in super-enhancers [65]. In addition, depletion of SNF5, a core subunit in the cBAF and the PBAF, increased the interaction of c-myc with its targets on the chromatin and enhanced the activity of c-myc [46]. Oncogenic AP-1 transcription factors are another group of proteins whose activity is selectively maintained after the loss of SNF5 expression [46]. Results from chromatin accessibility analysis in lung adenocarcinoma cells from a genetically engineered mouse model (GEMM) of Kras LSL-G12D/+ and Trp53 fl/fl (KP) initiated with BRG1 showed that metastasis-derived tumor cells were enriched for peaks with AP-1 transcription factor motifs, while other tumor cells were depleted of AP-1 peaks, suggesting that AP-1 may be involved in the metastasis of the tumor [66]. Furthermore, the inactivating mutation of ARID1A also reactivates the repressed TERT transcriptional activity and renders growth advantage to cancer cells [54].

### 5.3. Impairment of DNA Damage Repair Pathways and Genomic Instability by the Dysfunction of SWI/SNF Complexes

DNA is damaged by various endogenous and exogenous toxic chemicals. Double-strand breaks (DSBs) are one of the most deleterious DNA lesions, with serious consequences if they are not repaired. Therefore, multiple DNA repair pathways exist to repair DSBs. The contribution of defective DSB capacity to tumorigenicity has been widely recognized. There is plenty of research data supporting the theory that dysfunction of SWI/SNF complexes can impair DNA damage repair ability and might contribute to tumorigenesis. Multiple studies have shown that nucleosomes can block nucleases at DNA ends [67,68] and that alteration of SWI or SNF will hinder the access of nucleases to the DNA, which is essential for DNA damage repair. For example, suppression of ARID1A reduces both non-homologous end joining (NHEJ) and homologous recombination (HR) repair pathways [69,70]. A study by Park et al. showed that dysregulation of SMARCA4 results in inefficient DNA double-strand break (DSB) repair and a defect in γ-H2AX phosphorylation after DNA damage, suggesting that the SWI/SNF complexes facilitate DSB repair, at least in part, by promoting H2AX phosphorylation on chromatin [71].

In addition, ADID1A promotes mismatch repair (MMR) by recruiting MSH2 [72], which is one of the MMR proteins. MMR is a system for recognizing and repairing DNA damage during DNA replication. Cells with an impairment in DNA MMR due to the loss of ADID1A usually have high TMB.

Furthermore, ARID1A is also required to resolve transcription–replication conflicts. Otherwise, replication stress will ensue with an ARID1A deficiency. Activation of ATR (ataxia-telangiectasis-mutated and RAD3-related) and its downstream kinases, checkpoint kinases 1 and 2, will follow. Eventually, the replication cycle will be paused to resolve the conflict. Loss of ARID1A will cause the cells to become addicted to ATR activity [73].

Loss of SMARCA4 and ARID1A also impairs the function of topoisomerase II-alpha (TOP2A) and its crucial role in the decatenation of newly replicated sister chromatids during mitosis, which could also partially explain the high occurrence of mutations and high genetic instability in tumors harboring inactivating mutations of BRG1 and ARID1A.

Genomic instability in NSCLC with a deficiency of SWI/SNF is also related to enhanced Aurora A activity, which is one of the group kinases with serine/threonine activity and plays a crucial role in spindle assembly machinery during cell mitosis [74]. For example, Tagal et al. found that inactivation of AURKA induces apoptosis and cell death in vitro and in xenograft mouse models of NSCLC cells [75].

## 6. Vulnerability of NSCLC with Dysregulation of SWI/SNF Complexes

Inhibitors targeting synthetic lethality have been explored for therapeutic purposes in NSCLC treatment [73,76,77,78,79]. Synthetic lethality occurs when the inhibition of two genes is lethal while the inhibition of each single gene is not. The advancement of biological techniques for gene knockout has greatly accelerated the process of the identification of synthetic lethality with the loss of SWI/SNF complex genes.

EZH2 (enhancer of zeste homolog 2) is one of the most promising synthetic lethal targets identified with loss of SWI/SNF complexes [80]. EZH2, the catalytic subunit of polycomb repressive complex 2, silences gene expression through the methylation of lysine 27 on histone H3 (H3K27Me3) [53]. SWI/SNF and PRC2 complexes co-localize on the chromatin, antagonizing each other and playing opposite effects in the promotion of tumorigenesis [81]. Inactivating mutations of SWI/SNF derepress the activity of PRC2, and the cell adapts to the heightened PRC2 status. Recently, tazemetostat, the first oral EZH2 inhibitor, received FDA approval for patients with relapsed or refractory follicular lymphoma and advanced epithelioid sarcoma [82]. Various other EZH2 inhibitors are under clinical development, and there has been significant interest in combining EZH2 inhibitors with ICIs to overcome immunotherapy resistance by reprogramming the TME [83,84,85].

Another group of synthetic lethality targets is the mutually exclusive paralogs of the SWI/SNF complex subunit [86]. SMARCA4-deleted cancer cells are highly dependent on the paralog SMARCA2 for their survival, and ARID1B is required for the survival of ARID1A-depleted cells as well [13,76]. The ATPase domain and bromodomain in the BRG1 and BRM, as well as in the PMRM1, can be potentially used as druggable pockets to design small-molecular inhibitors. However, ARID domains containing SWI/SNF subunits, such as ARID1A, ARID1B, and ARID2, do not contain similar druggable pockets. The recently developed proteolysis-targeting chimera (PROTAC) technique circumvents the requirement for druggable pockets to target a gene [87]. PROTAC are pharmacological agents linking a binding ligand for the targeting gene with an E3 ubiquitin–protein ligase moiety. Different from other druggable pocket-based small-molecular inhibitors inhibiting the function of their target proteins, PROTACs transfer the ubiquitin onto the target protein first and initiate proteasomal degradation. For example, PRT3789 is a potent and selective BRM-targeted degrader. Preclinical experiments with PRT3789 demonstrated robust inhibition of cell proliferation in SMARCA4-deleted NSCLC in vitro and in PDX tumors ex vivo [88]. PRT3789 will soon be put into a phase I clinical trial for advanced or metastatic solid tumors [89].

In addition, dysfunction of ARID1A impairs multiple pathways functioning for DNA damage repair, such as DSB repair, MMR, and the resolution of stress induced by the transcription–replication conflict, etc. To compensate for the compromised DNA damage repair, the cancer cells with ARID1A deficiency have to depend on other redundant DNA repair pathways, such as poly-ADP ribose polymerase (PARP), to survive. It has been shown that NSCLC cells deficient in ARID2 are sensitive to the treatment of PARP inhibitors [90]. Comparably, cancer cells with ARID1A deficiency are also susceptible to the inhibition of ATR activity.

The significantly lower expression of cyclin D1 in BRG1-defient NSCLC cells compared with BRG1-intact normal control cells makes cyclin D1 as well as CDK4/6 proteins potential synthetic lethal targets for the treatment of NSCLC [91].

Finally, it was found that BRG1-deficient NSCLC cells have a reduced transcriptional response to energy stress and depend more on the oxidative phosphorylation pathway as their energy source. Thus, the oxidative phosphorylation pathway becomes a synthetic lethality target. IACS-010759, a potent inhibitor of the mitochondrial complex in the electron transport chain, has been shown to have an inhibitory effect on the growth of NSCLC cells [92].

Although there is no direct evidence to support the clinical use of these therapeutics targeting the vulnerabilities in NSCLC with SWI/SNF deficiency and the available data are mainly from the early stages of preclinical and phase I/II clinical studies in the general population of patients with NSCLC, we believe that data from many new studies will be pouring in soon about the efficacy and toxicity of the therapeutics for the treatment of the subset of NSCLC with SWI/SNF deficiency. As many as 32 clinical trials were identified by searching the clinicaltrials.gov website for therapeutics for the treatment of NSCLC patients with SWI/SNF deficiency (Table 5). Most of the studies are in the stage of recruiting study subjects or are in preparation for the recruitment of study subjects. The results of these studies should be beneficial to understanding the clinical value of the subset of NSCLC patients with a deficient SWI/SNF complex.

## 7. SWI/SNF Deficiency Influences the Immunogenicity of Malignancies

Immunotherapy with ICIs has been one of the standards of care for patients with advanced or early-stage NSCLC who underwent surgery, concurrent chemotherapy and radiation treatment. Six ICIs have been approved by the FDA for NSCLC so far (Table 1). However, a significant portion of patients either do not respond or respond initially but then progress due to the development of acquired resistance [93]. Understanding the mechanisms of the cancer cells to avoid ICI-mediated T-cell cytotoxicity and identifying the crucial regulators of the process are urgently needed to enhance the efficacy of cancer treatment with ICIs.

Dysfunction of the SWI/SNF complex occurs commonly in cancers of various histologies. Results from previous studies suggested that loss of SWI or SNF had a significant effect on the response and resistance of cancer patients to the ICI treatment.

The influence of the SWI/SNF complex on tumor immunity is not only dependent on its role in regulating gene transcription but also on its cooperation with other epigenetic regulators, especially PRC 2. Thus, the SWI/SNF complex can modulate tumor immunogenicity through multiple mechanisms.

Intrinsically, as a master regulator of gene transcription, deficiency of the SWI/SNF complex increases the expression of some isoforms of normal protein, which can function as neoantigens, thus increasing the possibility of tumor cells being recognized by an activated immune system. The SWI/SNF complex also plays an important role in the development of T lymphocytes and is crucial for the maturation of effector T lymphocytes [94,95,96]. Furthermore, SMARCA4 can upregulate the transcriptome of B cells during B-cell activation to promote cell proliferation [97]. For example, ARID1A deficiency will compromise the mismatch repair pathway, resulting in increased genomic instability and high TMB in the cell. PD-L1 protein was also highly expressed in ARID1A-deficient tumor cells [98], which predicts the level of the response to ICI treatment in lung cancer [16].

Inactivation of three members of the PBAF subfamily, PBRM1, Arid2, and Brd7, rendered mouse melanoma cells more sensitive to T-cell-mediated cytotoxicity in vitro and to ICI treatment in a mouse model in vivo [99]. A loss-of-function mutation in PBRM1 in kidney cancer was also associated with a better treatment response to PD-1 blockade [100].

As a non-covenant chromatin regulator, the SWI/SNF complex can cooperate with other epigenome regulators, such as PRC2, to maintain the landscape of the epitome of a cell. PRC2 is the covenant epigenetic regulator that deposits methyl groups onto the lysine 27 on histone H3 (H3K27mes) and represses its activity. Among the long list of gene targets under the repressive control of PRC2 are hundreds of IFN-γ-stimulated genes, cytokines, and receptors. The number of genes will be much greater in the cancer cells, specifically in the cancer cells with dysregulated SWI/SNF complexes, since the activity of PRC2 will be greatly elevated in the absence of the competition of the SWI/SNF complexes [101]. For example, in a tumor cell without the expression of ARID1A, the elevated methyl transferase activity of EZH2 will suppress the function of Th1-type chemokines and IFN-g-responsive genes by converting H3K27 to H3K27me3 on the Th1-type chemokines and the IFN response promoters. This results in limited CD8+ T-cell infiltration into the TME and a low immune response after treatment with ICI [102].

With regard to the immune microenvironment, SWI/SNF complexes are required for the expression of a large number of interferon (IFN)-inducible genes [103], including the induction of CIITA, the master regulator of major histocompatibility complex class II expression, which is essential for the induction of an effective immune response towards the tumor antigen [104].

SMARCA4 deficiency has been associated with elevated levels of tumor-infiltrating lymphocytes (TILs) [79,105], while ARID1A alterations were correlated with markedly high immune infiltrates in endometrial, stomach, and colon cancer but lower CD8+ T-cell infiltrations in ARID1A-mutant renal clear-cell carcinoma, indicating that the association between ARID1A alterations and immune infiltrates was cancer-dependent [31]. Adding another layer of complexity, a recent study reported that SMARCA4-deficient tumors were infiltrated by FOXP3+ (Forkhead box protein P3+) cells and neutrophils [106]. Both types of the two immune cells are important for the maintenance of an immunosuppressive environment favoring tumorigenesis [107,108].

**Figure 1:**
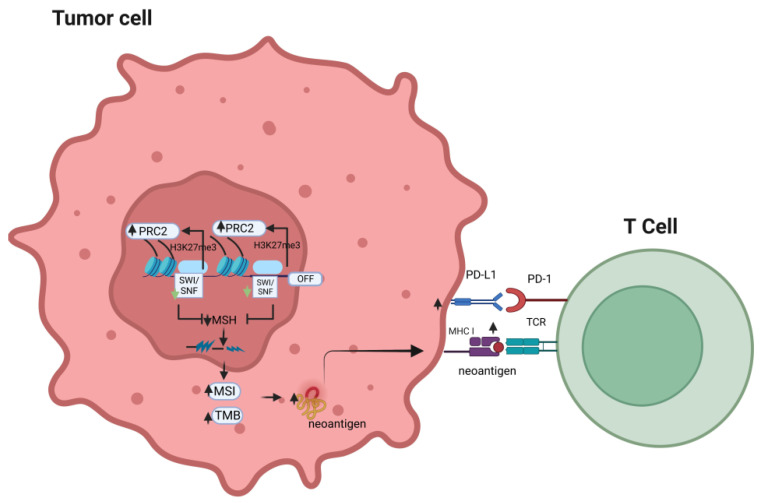
Effects of alteration of the SWI/SNF complex on the response to immune checkpoint inhibitors.

Therefore, the effects of dysregulated SWI/SNF on tumor immunogenicity combine with other genetic/epigenetic factors and tissue/differentiation-specific factors to determine the final result of the response to different combinations of ICI treatment, either monotherapy or combined therapy (Figure 1).

An alteration in the SWI/SNF chromatin remodeling complex may compensatorily elevate PRC2 activity, which results in decreased expression of the mismatch repair gene, MSH2. This may lead to increased tumor mutational burden and microsatellite instability, which will further promote the chance of neo-antigens to be presented by MHC class I and, in turn, enhance T-cell recognition of the tumor antigen, potentially sensitizing the tumor cells to the anti-tumor effects of immune checkpoint inhibitors (PRC2: polycomb-repressive complex; MSI: microsatellite instability; TMB: tumor mutation burden; TCR: T-cell receptor; MSH: mismatch repair gene).

## 8. Outcome of ICI Treatment for NSCLC with Deficiency of SWI/SNF

Deficiency of the SWI/SNF-complex impairs the ability of the cell to self-renew and differentiate. It is not surprising that NSCLC with loss-of-function mutations usually pursues an aggressive clinical course with high heterogeneity and eventually ends with a very dismal outcome [109]. To further explore the relationship between the deficiency of the SWI/SNF complex and response to the ICI treatment, we investigated the available relevant studies, as listed in Table 6. Table 6 lists the studies with the specification of their targeting subunits in the SWI/SNF complex and the outcome of the study. It is noteworthy that all the listed studies were retrospective except for one case report.

Results from a few studies suggested that NSCLC patients with a deficiency of SWI or SNF might be more sensitive to ICI treatment [109,110,112,113,114,115,117].

After reviewing these studies, it was found that results from the relationship between the deficiency of the SWI/SNF complex and response to the ICI treatment is inconsistent, with some showing a positive association and others showing a negative association.

In 2019, Naito T. reported that nivolumab could effectively reduce the metastasis of an SMARCA4-deficient lung adenocarcinoma for more than 14 months after the failure of three standard chemotherapy regimens. The tumor has a high TMB but no expression of the PD-L1 protein [113]. The results of another study carried out by Schoenfeld also support the idea that BRG1 can be a biomarker for ICI response. In the latter study, BRG1 deficiency was correlated with a higher overall response rate (ORR), accompanied by higher TMB, and lower PD-L1 expression compared with control patients [112]. However, this conclusion was not supported by the studies published by Dagogo Jack et al. and Alessi et al. [111,116]. One common factor in these two studies was that co-mutated genes were involved, where co-mutation of STK11/KEAP1 with BAG1 existed in all four cases in the study by Dagogo Jack and co-mutation of KRAS with BAG1. It is, therefore, reasonable to surmise that co-mutated genes need to be excluded before an SWI or SNF subunit can be evaluated as a biomarker for ICI treatment. The result from the study by Zhou et al. corroborated the assumption that the correlation between the alteration of BRG1 and longer progressive-free survival (PFS) was only present in the presence of “pure” alterations of BRG1 without STK11 or KEAP mutations [115,117]. It is well known that NSCLC cells harboring mutations of KRAS, P53, KEAP1, and STK11 are resistant to the ICI treatment [120]. Importantly, these genes are often highly mutated in NSCLC cells containing a deficiency of the SWI/SNF complex as well [100]. Thus, the value of the SWI/SNF complex deficiency as a biomarker of the ICI treatment for NSCLC should only be evaluated after excluding the influence of these co-mutated genes. Otherwise, the correlation disappeared or was minimized by the co-mutated genes.

High heterogenicity in the tumor with BAG1 deficiency [121] might also be responsible for the seemingly conflicting results of ICI treatment in NSCLC patients with alterations of SWI or SNF.

There are two studies assessing the relationship between loss of PBRM1 and response to ICI treatment. Results from the two studies consistently supported the notion that deficiency of the PBRM1 subunits might be a negative biomarker for the ICI response [118,119]. Interestingly, results from two reports about the relationship between deficiency of ARID subunits and response to ICI treatment showed that there was a positive correlation with higher median overall survival (MOS) and overall survival (OS) in NSCLC, with mutations of ARID1 accompanying a higher TMB and higher PD-L1 expression [115,117]. These results suggested that the alteration of different SWI/SNF subunits might have a unique impact on the response of NSCLC with deficient SWI/SNF subunits. Because the majority of the studies listed are retrospective and this may lead to biased conclusions, prospective studies among NSCLC patients are urgently needed to help answer this important question regarding the impact of an alteration in SWI or SNF on the response of a subset of patients to ICI treatment

## 9. Perspectives

Mutations of genes encoding for SWI/SNF chromatin remodelers occur commonly in NSCLC. Treatment of the subset of NSCLC with an alteration of SWI/SNF, which tends to be dedifferentiated with a very poor prognosis, has been explored extensively in recent years.

Certain synthetic lethality in these NSCLC cells can be exploited as vulnerable targets for their treatment. While many therapeutics targeting these vulnerabilities, such as EZH2 inhibitors and PARP inhibitors, have been approved by the FDA, some of them, such as PROTACs and ATR inhibitors, are still under clinical development (Kymera Therapeutics, Inc., Watertown, MA, USA, Impact Therapeutics, Inc., Nanjing, China).

Several reasons might contribute to the inconsistency in the results of studies about the relationship between the deficiency of the SWI/SNF complex and the response to the ICI treatment. Firstly, the response to ICIs might be confounded by the coexistence of mutations in other important tumor suppressors or oncogenes, such as co-mutated STK11, KEAP1, and P53, which can influence patients’ response to the treatment with ICIs. Secondly, high heterogenicity in the tumor with SWI/SNF deficiency might also be responsible for the seemingly conflicting results in the studies about the response to ICI treatment in NSCLC patients and alterations in SWI/SNF. In addition, an alteration in each different SWI/SNF subunit might have unique effects on the response of NSCLC with deficient SWI/SNF subunits to treatment with ICIs. Results from a future prospective study using a larger cohort of NSCLC patients will be needed to resolve the inconsistency about the issue.

Lastly, it is worth pointing out that combining inhibitors of other chromatin modulators with ICIs might be effective for the treatment of NSCLC with deficient SWI/SNF chromatin remodelers. As mentioned above, the elevated EZH2 activity in the tumor cell with the alteration of SWI and SNF subunits suppresses the function of Th1-type chemokines and IFN-g-responsive genes. One possibility is that the uncontrolled PRC2 activity may be partially responsible for the cold immunity in some SWI/SNF-deficient tumors, even though TMB was peculiarly high in some of these tumors [118,119]. Numerous studies have been carried out to test combination therapy to overcome the resistance of immunotherapy. Targeting SWI/SNF chromatin regulators can be a tantalizing strategy given the fact that SWI/SNF complexes might have a global effect on TME. The caveat is that the effect of these drugs targeting chromatin regulators might have heterogenous effects on various subsets of immune cells, so that the net effect of immune cell killing can be heterogenous and difficult to predict. We may be able to design a study using a new approach of antibody–drug conjugates [122] to target chromatin regulators on specific subsets of cells, and we hope that current and future clinical studies will shed light on this conundrum in the immunotherapy of NSCLC patients with SWI/SNF deficiency.

## Figures and Tables

**Table 1:** List of Immune checkpoint inhibitors approved by FDA for the treatment of NSCLC. Ref. [9].

Name	Target	Year of Approval	Approved Clinical Indication
Nivolumab	PD-1	2022	Nivolumab + chemotherapy as neoadjuvant treatment
		2020	Nivolumab + ipilimumab + limited chemotherapy as 1st treatment of metastatic or recurrent NSCLC
		2020	Nivolumab + ipilimumab as 1st treatment of Metastatic NSCLC (PD-L1 ≥ 1%)
		2015	Advanced (metastatic) NSCLC progressed during or after platinum-based chemotherapy.
		2015	Advanced (metastatic) squamous NSCLC with progression on or after platinum-based chemotherapy.
Pembrolizumab	PD-1	2018	Pembrolizumab +chemotherapy for the 1st treatment of metastatic squamous NSCLC
		2017	Pembrolizumab + chemotherapy for the 1st treatment of metastatic non-squamous NSCLC, ± PD-L1
		2016	Metastatic NSCLC (PD-L1 ≥ 50%)) without EGFR /ALK genomic tumor aberrations
		2015	Advanced NSCLC progressed after other treatments and with tumors that express PD-L1
Cemiplimab	PD-1	2022	Cemiplimab + chemotherapy as 1st treatment for advanced NSCL
Atezolizumab	PD L-1	2021	Adjuvant treatment following surgery and chemotherapy for stage II-IIIA NSCLC (PD-L1 ≥ 1%)
		2020	1st treatment for NSCLC with PD-L1 expression no EGFR/ALK genomic tumor aberrations
		2019	Atezolizumab + chemotherapy for the 1st treatment of NSCLC no EGFR/ALK aberrations.
		2018	Atezolizumab +bevacizumab+chemotherapy 1st treatment of metastatic NSCLC no EGFR/ALK aberrations
		2016	Metastatic NSCLC who have disease progression during/following chemotherapy
Durvalumab	PD L-1	2022	Durvalumab + Tremelimumab + chemotherapy for the treatment of metastatic NSCLC
		2018	Unresectable stage III not progressed NSCLC after treatment with chemotherapy and radiation
Ipilimumab	CTLA-4	2020	Ipilimumab+nivolumab + chemotherapy as 1st treatment of metastatic or recurrent NSCLC
		2020	Ipilimumab + nivolumab as 1st treatment of NSCLC (PD-L1 ≥ 1%)
Tremelimumab	CTLA-4	2022	Tremelimumab + Durvalumab+chemotherapy for the treatment of metastatic NSCLC

**Table 2:** List of epigenetic agents approved by FDA. Refs. [17,26].

Name	Year of Approval	Target	Clinical Use
Azacitidine	2004	DNMT	Myelodysplastic syndrome
Decitabine	2006	DNMT	Myelodysplastic syndrome
Hydralazine	1953	DNMT	Hypertension
Belinostat	2014	HDAC	Peripheral T-cell lymphoma
Panobinostat	2015	HDAC	Multiple myeloma
Vorinostat	2006	HDAC	Cutaneous T-cell lymphoma
Romidepsin	2009	HDAC	Cutaneous T-cell lymphoma
Enasidenib	2017	IDH2	Acute myeloid leukemia
Ivosidenib	2018	IDH1	Acute myeloid leukemia
Tazemetostat	2020	EZH2	Epithelioid sarcoma/follicular lymphoma

**Table 3:** Function and identified binding domains of SWI/SNF subunits.

Function	Subunit	Alias	BAF/PBAF/ncBAF	Mutual Exclusive Paralog	Domains
ATPase	SMARCA2	BRM	+/−/+ *	SMARCA2/A4	Bromodomain, HSA, SnAC
ATPase	SMARCA4	BRG1	+/+/+		Bromodomain, HAS, SnAC
Core	SMARCC1	BAF155	+/+/+	SMARCC1/C2	Chromodomain, SWIRM, SANT
Core	SMARCC2	BAF170	+/+/+		Chromodomain, SWIRM, SANT
Core	SMARCD1	BAF60A	+/+/+	SMARCD1/D2/D3	
Core	SMARCD2	BAF60B	+/+/+		
Core	SMARCD3	BAF60C	+/+/+		
Core	SMARCB1	BAF47	+/+/−		WH
Core	SMARCE1	BAF57	+/+/−		HMG
Accessory	BCL7A	BCL7A	+/+/+		
Accessory	BCL7B	BCL7B	+/+/+		
Accessory	BCL7C	BCL7C	+/+/+		
Actin	ACTL6A	BAF53A	+/+/+	ACTL6A/6B	
Actin	ACTL6B	BAF53AB	+/+/+		
Actin	ACTB	-ACTIN	+/+/+		
Accessory	SS18	SSXT	+/−/+		
Accessory	SS18L1	CREST	+/−/+		
BAF unique	ARID1A	BAF250A	+/−/−	ARID1A/1B	ARID, ARM, ZNF
BAF unique	ARID1B	BAF250B	+/−/−		ARID, ARM, ZNF
BAF unique	DPF1	BAF45B	+/−/−	DPF1/F2/F3/	PHD finger
BAF unique	DPF2	BAF45C	+/−/−		PHD finger, ZNF
BAF unique	DPF3	BAF45BD	+/−/−		PHD finger, ZNF
PBAF unique	PHF10	BAF45A	−/+/−		PHD finger, ZNF
PBAF unique	PBRM1	BAF180	−/+/−		Bromodomain, BAH, HMG, ZNF
PBAF unique	ARID2	BAF200	−/+/−		ARID, WH, ZNF, ARM
PBAF unique	BRD7	BRD7	−/+/−		Bromodomain
ncBAF unique	BICRA	GLTSCR1	-/-/+		
ncBAF unique	BICRAL	GLTSCR1	−/−/+		
ncBAF unique	BRD9	BRD9	−/−/+		Bromodomain

* + − denote presence or absence in the SWI/SNF complex. Bromodomain recognizes acetylated lysine residues in histone; The helicase/SANT-associated (*HSA*) *domain* is a predicted DNA-binding domain. The Snf2 ATP coupling (SnAC) domain recognizes the acidic patch of the nucleosome; Swi3p, Rsc8p, and Moira (SWIRM) and Swi3, Ada2, N-CorR, and TFIIIB (SANT) domains recognize nucleosomal DNA; The winged helix (WH) domain is a DNA-binding domain. The HMG domain is a DNA-binding domain. AT-rich interactive domain (ARID) is one of the HTH-comprising DNA binding domains. Armadillo repeat (Arm) repeat domains are involved in protein-protein interactions. Plant homeodomain (PHD) fingers recognize methylated and unmethylated histone H3K4; The Bromo adjacent Homology (BAH) domain recognizes methylated histone and nucleosome binding; The zinc-finger domain (ZNF) binds to DNA, RNA, and proteins. Refs. [28,29,30,31].

**Table 4:** Frequency of SWI/SNF gene mutation in NSCLC and analysis of mutation types in the top six genes with highest mutation frequencies.

Subunit	Mutation Freq (%)	Misssense N (%)	Truncating N (%)	Inframe N (%)	Splice N (%)
SMARCA4.	7	344 (54.5)	206 (32.6)	10 (1.6)	71 (11.3)
ARID1A	6	271 (42.7)	322 (50.8)	6 (0.9)	35 (5.5)
ARID1B	4	279 (75.0)	70 (18.8)	13 (3.5)	0 (2.7)
ARID2	4	227 (52.8)	168 (39.1)	0 (0.0)	35 (8.1)
SMARCA2.	2.8	8 (5.8)	0 (0.0)	1 (0.7)	
PBRM1	2.5	126 (57.0)	64 (29.0)	1 (0.5)	30 (13.6)
DPF3	1.9				
SMARCC2	1.7				
BICRAL	1.7				
SMARCC1	1.6				
ACTL6B	1.4				
BICRA	1.3				
DPF2	1.2				
ACTB	1.1				
SS18	1				
ACTL6A	0.9				
BRD7	0.8				
BRD9	0.8				
SMARCD2	0.8				
DPF1	0.8				
SMARCD1	0.7				
SS18L1	0.6				
SMARCB1	0.5				
SMARCD3	0.3				
SMARCE1	0.3				
BCL7A	0.3				
BCL7B	0.2				
BCL7C	0.2				
PHF10	0.2				

Result from a combined 28 study with 8854 patients and 11037 samples in cBioPortal. 2201 (25%) of queried patients and 2404 (22%) of samples have mutations.

**Table 5:** Clinical trials with vulnerability targeting therapeutics for NSCLC with SWI/SNF deficiency.

Identifier	Status	Drugs	Target	Subject	Phase
NCT05467748	Not yet recruiting	* Tazemetostat + PD-1 mAb	EZH2	Progressed with an anti-PD-1/L1 mAb	Phase 1/2
NCT05639751	Not yet recruiting	PRT3789	BRM	Advanced solid tumor with loss of SMARCA4	Phase 1
NCT01082549	Completed	Chmo ± * Iniparib	PARP	Untreated NSCLC stage III	Phase 3
NCT04538378	Recruiting	* Olaparib + Durvalumab	PARP	EGFR Mutated NSCLC	Phase 2
NCT05127590	Recruiting	RBN-2397 + Pembrolizumab	PARP	Advanced Squamous NSCLC	Phase 2
NCT03330405	Not yet recruiting	* Talazoparib + Avelumab	PARP	Primary or Recurrent or Metastatic Solid Tumors	Phase 2
NCT04380636	Recruiting	Pembrolizumab+chmo	PARP	NSCLC stage III	Phase 3
		Pembrolizumab ± * Olaparib			
NCT01386385	Not yet recruiting	Veliparib ± Radiation Therapy	PARP	Stage III unremovable NSCLC	Phase 1/2
		Carboplatin/Paclitaxel			
NCT02944396	Completed	Veliparib + Nivolumab	PARP	Metastatic or Advanced NSCLC	Phase 1
		+Platinum			
NCT03308942	Completed	* Niraparib ± PD-1 mAb	PARP	Advanced NSCLC no chemo or PD-L/1/ mAb with high PD-L1	Phase 2
NCT02412371	Terminated	Veliparib + chemo	PARP	NSCLC stage III	Phase 1/2
NCT02264990	Completed	Veliparib + chemo	PARP	Non-squamous NSCLC	Phase 3
NCT02292550	Completed	* Ribociclib and Ceritinib	CDK4/6	ALK-positive NSCLC	Phase 1
NCT04863248	Terminated	* Trilaciclib + Docetaxel	CDK4/6	Metastatic NSCLC	Phase 2
NCT03455829	Completed	G1T38, + Osimertinib	CDK4/6	EGFR-Mutant NSCLC	Phase 1/2
NCT02022982	Not yet recruiting	* Palboclclb + PD-0325901	CDK4/6	KRAS Mutant NSCLC	Phase 1/2
NCT03170206	Recruiting	Palbociclib + MEK162	CDK4/6	Advanced KRAS Mutant NSCLC	Phase 1/2
NCT03965845	Completed	Palbociclib + Telaglenastat	CDK4/6	Solid tumor (including NSCLC)	Phase 1/2
NCT04545710	Recruiting	Osimertinib + * Abemaciclib	CDK4/6	EGFR Mutant NSCLC after Osimertinib	Phase 1
		Spartalizumab/* Ribociclib			
NCT03386929	Not yet recruiting	Avelumab/Axitinib/	CDK4/6	Advanced or metastatic NSCLC	Phase ½
		* Palbociclib			
NCT02664935	Not yet recruiting	Multi drug trial including	CDK4/6	NSCLC	Phase 2
		* Palbociclib			
NCT05538572	Recruiting	PRT3645	CDK4/6	Solid tumor (including NSCLC)	Phase 1
NCT04591431	Recruiting	Multi drug trial including	CDK4/6	Solid tumor (including NSCLC)	Phase 2
		* Palbociclib			
NCT04606446	Recruiting	Multi drug trial including	CDK4/6	Solid tumor (including NSCLC)	Phase 1
		* Palbociclib			
NCT05358249	Recruiting	JDQ443 + trametinib	CDK4/6	K-Ras G12C Solid tumor (including NSCLC)	Phase 1/2
		+* Ribociclib			
NCT04491942	Recruiting	BAY 1895344 + chemo	ATR	Advanced Solid tumor (including NSCLC)	Phase 1
NCT02264678	Recruiting	* Ceralasertib + chemo	ATR	Advanced Solid tumor (including NSCLC)	Phase 1/2
NCT01471964	Terminated	MLN8237 + Erlotinib	Aurora A	NSCLC	Phase 1/2
NCT05017025	Recruiting	LY3295668 + Osimertinib	Aurora A	Advanced or Metastatic EGFRMutant NSCLC	Phase 1/2
NCT05374538	Recruiting	VIC-1911 + Sotorasib	Aurora A	KRAS G12CMutant NSCLC	Phase 1
NCT01045421	Completed	Alisertib		Nonhematological Malignancies including NSCLC	Phase 1/2
NCT02635061	Not recruiting	ACY 241 + Nivolumab	HDAC6	Unresectable NSCLC	Phase 1

* FDA approved drugs. Information collected from clinicaltrials.gov.

**Table 6:** Impact of alteration of SWI/SNF on the outcome of ICI treatment in advanced NSCLC.

Type of Study	Number of Subjects	Targeting Subunits	Year of Publication	Clinical OUTCOMES	References
Retrospective	441	BRG1	2020	Longer PFS in NSCLC with ‘pure ‘alterations of BRG1 without STK11/KEAP mutations	[110]
Retrospective	11	BRG1	2020	4 NSCLC with BRG1 mutation ICI monotherapy, ¼ lost follow-up, ¾ primary progression	[111]
Retrospective	445	BRG1	2020	Higher ORR in NSCLC with BRG1 mutation, higher TMB, lower PD-L1 expression	[112]
Case report		BRG1	2019	Obvious reduction of metastasis for longer than 14 months	[113]
Retrospective	63	BRG1	2022	Shorter OS, increased FOXP3+ and neutrophil but no CD8 + cells	[104]
Retrospective	146	BRG1/ARID1A	2022	Longer PFS in NSCLC with alterations of BRG1/ARID1A	[114]
Meta study	3416	ARID1A/1B/ARID2, BRG1	2021	Longer mOS NSCLC with mutations of ARID1A/1B/ARID2, higher TMB	[115]
Retrospective	136	ARID1A/1B/ARID2, BRG1, PBRM1	2021	Shorter OS in NSCLC with BAG1 and K-RAS co-mutation, higher TMB	[116]
Retrospective	240	ARID1A/1B	2020	Longer OS and PFS in NSCLC with ARID1 mutation, higher TMB, higher ratio of PD-L1	[117]
Retrospective	350	PBRM1	2022	Shorter OS and PFS in NSCLC with PBRM1 mutation	[118]
Retrospective	441	PBRM1	2020	Shorter OS	[119]

OS: overall survival. mOS: median overall survival. PFS: progression free survival. ORR: overall response rate. Highlighted: positive correlation between alteration of SWI/SNF and outcome of ICI treatment. of SWI/SNF and outcome of ICI treatment.

## Data Availability

Not applicable.
